# Availability of psychological therapies and workforce participation of individuals with long-term mental health problems: a retrospective observational study

**DOI:** 10.1186/s13033-026-00706-z

**Published:** 2026-04-15

**Authors:** Joe Dodd, Igor Francetic, Peter Bower, Jon Gibson

**Affiliations:** 1 Centre for Primary Care and Health Services Research, the University of Manchester, Manchester, M13 9PL UK; 2 Department of Business Economics, Health and Social Care, University of Applied Sciences and Arts of Southern Switzerland, Manno, Switzerland

**Keywords:** Mental health, Workforce participation, Health policy, Healthcare access, Psychological therapy interventions

## Abstract

**Background:**

In response to the global prevalence and societal impact of mental health problems, innovative healthcare policies have improved access to psychological therapy interventions. Yet, the indirect effects of these access policies on labour force participation gaps related to mental health problems remain unclear. This study assessed the relationship between one of the first major policies to improve access to psychological therapies, the NHS Talking Therapies service, and the economic activity of individuals with long-term mental health problems.

**Methods:**

In this retrospective observational study, we derived a national sample of the English working-age population from Annual Population Survey data for the period 2015–2020 (*N* = 535068). Data included information on economic activity and health status, but not the use of healthcare services. The outcome of the study was labour force participation. The volume of appointments per referral received by NHS Talking Therapies services across healthcare commissioning regions in England was used to measure the regional supply of psychological therapy interventions. We used linear regression models, adjusting for a comprehensive set of individual and area-level controls, to estimate the association between the regional supply of psychological therapy interventions and labour force participation for those with and without a long-term mental health problem at the population level.

**Results:**

We find a labour force participation gap of 36% between individuals reporting long-term mental health problems and otherwise similar individuals with no reported mental health problems. Holding all else equal, we find an increase in the regional supply of NHS Talking Therapies of one appointment per referral is associated with a 0·92 percentage point (CI: 0·0018 − 0·0165) reduction in the probability of labour force participation gap. Results of sub-sample analyses suggest this association was driven by individuals who were not claiming benefits, aged between 45 and 65, and reported male gender.

**Conclusions:**

Policymakers should consider the indirect effects of policies that improve access to psychological therapy interventions as a potential moderator of the labour force participation gap related to long-term mental health problems.

**Supplementary Information:**

The online version contains supplementary material available at 10.1186/s13033-026-00706-z.

## Background

In response to the high prevalence and impact of mental health problems, research has supported the development of pharmacological and psychological therapy interventions for mental health disorders, which are clinically effective at symptom reduction, and also demonstrate indirect gains in outcomes such as increased labour market productivity (lower absenteeism and presenteeism) and labour force participation [1–5]. To deploy these evidence-based interventions at scale, policymakers implemented a range of innovative healthcare policies that improve access to them. Launched in 2008, the English National Health Service (NHS) Talking Therapies for Anxiety and Depression model (hereby referred to as NHSTT) is a policy aimed at improving access to evidence-based psychological therapy interventions, and is often considered the first of its kind and scale globally [6]. In the NHSTT service model, referrals are placed on a waiting list for a face-to-face or telephone assessment appointment by a mental health practitioner before they are offered treatment. During the assessment appointment, the patient will be informed about the intervention options tailored to their goals and needs, the anticipated waiting time before starting treatment, and the number of treatment appointments available per course of treatment. Once treatment begins, the patient will be offered additional treatment appointments (up to a limit depending on the severity of symptoms/condition) on a session-by-session basis, managed using routine outcome measurements, which include specific questionnaires designed to quantify the impact of symptoms related to a particular mental health problem, such as the PHQ-9 and the GAD-7. As such, the supply of services and availability of appointments may impact the number of treatment sessions offered to a patient.

The integration of the NHSTT service into primary care networks increased the provision of psychological therapy interventions for use with or without concurrent medication treatment and has become the main treatment pathway for common mental health problems in England. NHSTT operates with the objectives of continuously expanding the supply of mental healthcare, removing barriers to accessing its services, reducing stigma surrounding mental health problems, increasing awareness of NHSTT services in local populations, and supporting patients to find and remain in work [7]. Clinical studies have demonstrated that NHSTT treatment can cost-effectively improve patient recovery from symptoms of mental health problems [8].

Our study aims to assess how the regional supply of NHSTT relates to the economic activity of individuals with long-term mental health problems. The impact on daily functioning caused by long-term mental health problems lowers individual-level productivity and earnings potential, creating harmful labour force participation gaps (exits from the workforce) that snowball into significant societal costs consisting of lower aggregated productivity, lost tax revenue, welfare payments to support those in income poverty, and increased risk of income poverty in later life [9–16]. To unpick the association between regional supply of NHSTT and economic activity, we claim that the regional variation over time in the supply of NHSTT in England is plausibly exogenous from the perspective on individuals. Variation in supply is caused by the regional commissioning of healthcare by local governing bodies based on estimations of their population’s need for treatments [7, 17].

A direct effect on economic activity could arise from individuals who utilised the supply of NHSTT, as studies of administrative records have found an increased probability of employment in short and long-term follow-up periods for NHSTT patients who were out of the labour force due to illness at the start of treatment [18, 19]. However, there are well-established low rates of NHSTT treatment uptake and engagement among individuals with mental health treatment needs [20–23]. Recent evidence estimates that between 2008 and 2018, only a fifth of all working-age patients with a mental health diagnosis went on to have a course of NHSTT treatment [24]. We therefore posit that the regional supply of services providing psychological therapy interventions, strongly integrated into primary care networks with operating objectives to reduce stigma and increase awareness of mental health problems, could have indirect effects on the economic activity of local populations. One possible effect of NHSTT supply is an improvement in help-seeking behaviour, as this is predicted by mental health literacy, attitudinal and structural barriers to access, the acceptability and availability of treatments, and financial and time costs [25–34]. An improvement in help-seeking behaviour could lead to improved uptake and use of NHSTT treatment, improving quality of life and capacity to work. Another possible effect relates to the impact of NHSTT supply on mental health stigma in an area. Segregation and segmentation forces within the UK labour market, revealed through analysis of employment outcomes during the COVID-19 pandemic, suppressed the ability of individuals with a long-term mental health condition to find jobs that accommodate their needs [15]. Productivity losses attributed to long-term mental health problems arise when these individuals interpret the segregation and segmentation forces as a lack of available supportive roles, leading to exits from the workforce. If stigma is reduced in areas of high supply, we may observe a smaller labour force participation gap attributable to mental health problems. Additionally, indirect effects may occur through local healthcare management of mental health problems. For example, the Goldberg-Huxley pathway to psychiatric care model demonstrated that the availability of treatments previously affected General Practitioners’ (GPs; family doctors in the NHS healthcare system) referral and management of patients with mental health problems [32, 33]. A higher frequency of engagement with NHSTT services could influence GPs to lower their thresholds for referral to NHSTT treatments and/or use their freed-up resources to treat other patients with mental health problems themselves.

## Methods

### Data

For this retrospective observational study, we pooled all available data on English residents aged 18–65 collected by the UK’s Annual Population Survey (APS) between April 2015 and March 2020. This is a continuous household survey with a repeated national cross-sectional study design, managing a target number of economically active participants per governing region boundary [35]. The APS follows the International Labor Organization definition of economic activity, meaning economically active participants were individuals available to work in the short term, regardless of whether they were employed [36]. The study period was chosen as the sampling boundaries closely aligned with NHS healthcare commissioning regions during this time, and to avoid changes in APS data collection procedures resulting from the COVID-19 pandemic. More detailed information on the APS sampling and interview procedures and how targets of economically active participants are met, along with descriptions of governing and commissioning regions in England, are provided in an additional file (see Additional File 1).

### Labour force participation

The primary outcome of our study was individual-level probability of labour force participation, represented by a binary variable taking a value of one for those in the labour force and zero otherwise. Participant information captured by a derived variable in the APS data was used to sort individuals into the outcome categories. The derived variable compiles the responses to a set of questions that comprehensively capture the International Labor Organization definition of economic activity to ascertain whether an individual was in work or available for work (labour force participants; employed and unemployed), or unavailable for work (out of the labour force), the week before the survey interview was completed. By this definition, individuals who were unemployed and available to work in the past four weeks and the next two weeks from the reference point were labour force participants. We use unavailability to work categories to exclude participants we assume had inelastic labour supply at the extensive margin and were therefore unlikely to be affected by the availability of mental healthcare. The excluded categories include students, those who are retired, and those who answered ‘other reason’ or did not provide a reason for unavailability to work in the previous four or next two weeks at the time of completing the survey. The derivation of this variable and proportions of the starting sample for each economic activity category, including how they are considered in the outcome, are presented in an additional file (see Additional File 2).

### Supply of NHS talking therapies

We use two sources of data to construct a regional-level NHSTT supply measure: NHS-produced administrative data reports on NHSTT activity data (further information provided in Additional File 3) and annually updated health geography area boundaries for assigning APS participants to their corresponding commissioning region [37–43]. During our study period, commissioning regions were Clinical Commissioning Groups (CCGs). CCGs received a budget to commission healthcare services for the local population, determined by an age- and deprivation-weighted capitation formula due to differences in population size and demographics across the areas they serve [44]. In total for our study period, there were 219 different CCG regions (217 in 2015/16, and 191 in 2019/20), with an estimated average of 304296 registered patients per region (minimum 74028, maximum 1320168), and an estimated average funding allocation of £1250 per patient in a region (minimum £946, maximum £1608) [44].

In our study, regional NHSTT supply is represented by the number of NHSTT appointments offered to patients per referral received. In the administrative data reports, appointments offered to patients include all care contacts with patients referred to a service within a commissioning region (assessment, treatment, reviews, employment advice), and the referrals received represent the demand for services in the region from all intake pathways (self, GP, and so on), for a given month. NHSTT services were commissioned through block contracts, whereby a provider or group of providers receives a lump-sum payment upfront to manage the provision of NHSTT treatments in a commissioning region for a given period (quarterly or annually). Block contracts typically reflect the expected patient demand for the service and previous expenditure on treatments. Therefore, service providers in a commissioning region had a fixed budget to allocate towards the different costs of providing treatments (staffing, rent and rates, technology/online platforms for treatment) that must meet the care and quality standards the NHS sets out for the service. These providers would seek to maximise the supply of appointments they provide, given the constraint of a fixed budget and patient demand, to maintain their contracts. We choose this measure of supply as it captures how well providers within a region managed their budget to provide clinical care contract appointments, scaled by the demand for treatments in a commissioning region, which would plausibly relate to individuals’ help-seeking behaviour and GP management of mental health problems. We construct this measure by summing the number of appointments offered and dividing by the total referrals received in each commissioning region in England for the three months before the survey interview. The three-month period was chosen to capture the supply of NHSTT services in an APS participant’s commissioning region relevant to the reference period of the labour force participation outcome.

### Long-term mental health problem

The APS captures information on whether participants reported having a health problem that lasted or was expected to last longer than twelve months, and how these interact with a person’s ability to work and reported economic activity. We created a binary indicator taking a value of one if a long-term health problem is reported as depression, bad nerves, anxiety, or a mental disorder and zero otherwise. We used this variable to define groups of individuals in the APS sample with and without a self-reported long-term mental health problem. To note, the APS does not capture whether participants utilised NHSTT services. Further information on the APS health questions and their relation to the binary health problem indicator is provided in an additional file (see Additional File 4).

### Statistical analysis

We reported the baseline characteristics (counts and percentages or averages and standard deviations) of the analysis sample stratified by whether the individual reported a long-term mental health problem. We also present proportions (counts and percentages) of labour force participation and long-term mental health problems when stratifying the sample on whether an individual lived in a low or high NHSTT supply commissioning region. Regions were categorised as low or high supply if the NHSTT supply measure was less than or more than one standard deviation unit from the analysis sample average.

We aim to estimate the unbiased Intention-to-Treat (ITT) association between NHS Talking Therapies supply and the individual-level probability of labour force participation using a linear probability model specification. We chose a linear probability model over a non-linear model due to its ability to accommodate fixed effects that isolate and partial out the influence of unobserved factors related to the regional commissioning of NHSTT services and local labour markets. The ITT association is our chosen target parameter as we investigate the indirect effects of a policy to expand access to evidence-based psychological therapy interventions on all individuals who could benefit from an increased supply of mental healthcare services, not just those who used and completed NHSTT treatment. To assess the association between the regional supply of NHSTT and labour force participation, we fully interacted the NHSTT supply measure with an indicator for having a long-term mental health problem. Notably, the APS does not capture whether participants utilised NHSTT services. Our preferred adjusted model specification is presented in Eq. (1).

1 $$\begin{aligned} {E}_{i,r,t} & ={{\upbeta\:}}_{0}\:+{\beta\:}_{1}I\left\{MH{P}_{i,t}=1\right\} +{\beta\:}_{2}{Supply}_{i,r,t} \\ & \quad +{\beta\:}_{3}I\left\{MH{P}_{i,t}=1\right\}\times\:{Supply}_{i,r,t} \\ & \quad +{\beta\:}_{4}{Waitingtime}_{i,r,t}+{\beta\:}_{5}I\left\{MH{P}_{i,t}=1\right\} \\ & \quad \times\:{Waitingtime}_{i,r,t}+{\beta\:}_{6}{\boldsymbol{X}}_{i,t}+{\boldsymbol{R}\boldsymbol{e}\boldsymbol{g}\boldsymbol{i}\boldsymbol{o}\boldsymbol{n}}_{i,r} \\ & \quad \times {\boldsymbol{P}\boldsymbol{e}\boldsymbol{r}\boldsymbol{i}\boldsymbol{o}\boldsymbol{d}}_{i,t}+{\epsilon\:}_{i,r,t}\end{aligned}$$

where $\:{E}_{i,r,t}$ is our binary outcome reflecting whether individual *i*, residing in commissioning region *r*, actively participated in the labour force during the financial year and quarter *t*. $\:{\beta\:}_{0}$ reflects the estimated intercept of the Linear Probability Model. $\:I\left\{{MHP}_{i,t}=1\right\}$ is the indicator function depicting whether individual *i* reported a long-term mental health problem during the financial year and quarter *t*. $\:{Supply}_{i,r,t}$ represents the NHSTT regional supply measure. This is the sum of the number of appointments offered divided by the sum of referrals received in commissioning region *r* for the three months before individual *i* completed the survey in the financial year and quarter *t*. This interaction term allows us to assess the association between labour force participation and regional NHSTT supply attributable to long-term mental health problems, comparing otherwise similar individuals. We control for the leading three-month average of median waiting times from referral to starting NHSTT treatment, represented by the variable $\:{Waitingtime}_{i,r,t}$. This reflects the temporal distance to treatment found to influence the health-seeking behaviour of individuals with a need for mental health treatment [29]. During the study period, mental healthcare was commissioned regionally based on need, which was predicted through individual (personal demographics) and area-level (deprivation and economic opportunity) factors [32, 45]. Therefore, we adjust the model for the set of covariates $\:{\boldsymbol{X}}_{i,t}$, which includes age, gender, single marital status, education level, whether claiming benefits, number of dependent children in the household, self-reported long-term physical health problems, area-level deprivation. Area-level deprivation is measured by the Index of Multiple Deprivation (IMD), comprised of several domains such as employment prospects, access to healthcare, crime, housing, and so on. The ${\boldsymbol{R}\boldsymbol{e}\boldsymbol{g}\boldsymbol{i}\boldsymbol{o}\boldsymbol{n}}_{i,r}\times\:{\boldsymbol{P}\boldsymbol{e}\boldsymbol{r}\boldsymbol{i}\boldsymbol{o}\boldsymbol{d}}_{i,t}$ interaction in Eq. (1) reflects the high-dimensional Fixed Effect representing the commissioning region the participant resides in and the period (quarter and financial year) in which they completed the survey. The former captures broader regional specific differences, whereas the latter accounts for any observed and unobserved seasonal variation in NHSTT demand and supply factors entering the model from the clustered stratified APS sampling and NHS financial reporting schedule. Using interacted Fixed Effects creates a unique intercept for each time period of all commissioning regions recorded in the sample, capturing any interaction of time and region-specific trends in the supply of NHSTT or local labour markets. Assuming that the regional need for NHSTT treatment evolves smoothly across time periods, this statistical procedure isolates variation in supply in deviation from predicted regional need, which we claim to be as-good-as-randomly assigned from the perspective of individuals. A full description of all variables included in the analysis, along with further information on the model assumptions, is provided in an additional file (see Additional File 5).            

We conducted several additional analyses to assess the robustness of our main results. We estimated our model on the analysis sample stratified by the financial year in which the survey was completed to assess whether the main result remained constant over the study period. For comparability with previous studies, we estimated the preferred specification using an outcome variable indicating whether the individual was in paid work at the time of completing the survey. For this outcome, we move the ILO unemployed category of economic activity to the zero category of the labour force participation outcome, and then exclude from the analysis any individual who reported unpaid family work. As a falsification test, we estimated our preferred specification on two outcomes alternative outcomes. The first was an adjusted labour force participation outcome, in which we replaced the reasons for non-participation in the labour force with the excluded categories we assumed represented inelastic labour supply at the extensive margin, and would not likely be affected by the supply of NHSTT: students, those who were retired, and those who did not specify a reason for unavailability to work. The second was a binary outcome indicating whether an APS participant reported having religious beliefs at the time of completing the survey, which we suggest was not related to the commissioning of mental healthcare, nor the likelihood of labour force participation. We tested for the presence of multicollinearity in our model by regressing the NHSTT supply measure on the model covariates. Following this, we check for any impact of repeated APS participants in the analysis sample on our findings due to sampling structure of the two APS components (see Additional File 1), any sensitivity of the main results to more restrictive mental health problem indicators (is a participant’s main health problem and is work-limiting), and any confounding due to complex healthcare needs by expanding the interaction term to include the long-term physical health problem covariate.

We also assessed three potential sources of heterogeneity by estimating separate stratified models. The first relates to whether individuals claimed benefits. Individuals who claimed benefits to supplement or top up their income may have approached the decision to participate in the labour force differently from those who did not, and could be influenced less by the supply of NHSTT. The second stratification grouped individuals by age (18–44 or 45–65). Those aged 18–44 were beginning and advancing their career, whereas those aged 45–65 are more likely to be at the peak of their earnings potential and approaching the decision to exit the labour force through retirement. Thirdly, we check for a difference in estimates by gender; recent evidence of gendered treatment effects of psychological therapy interventions on employment outcomes reveals that individuals who identified as male may be affected more by the supply of NHSTT [46].

We estimate the adjusted model specifications using the Least Squares Dummy Variable method with absorption of the interacted commissioning region and time period fixed effects on the pooled cross-sectional samples for years 2015 to 2020. We report three Average Marginal Effects (AMEs) obtained from the regression results. The first and second AMEs capture the association between NHSTT supply and labour force participation for individuals who reported and did not report a long-term mental health problem, respectively, reflecting the adjusted average predicted probabilities of the supply measure on the outcome for each indicator level when model covariates were averaged across the entire sample. The third AME captures the adjusted average predicted probability of NHSTT supply on labour force participation separately for the long-term mental health problem indicator levels when model covariates were averaged within the indicator groups. This captures the association between NHSTT supply and any ‘gap’ in labour force participation observed when stratifying the sample by reporting of long-term mental health problems. We report AMEs with 95% confidence intervals estimated using the Delta method. Robust standard errors are clustered at the commissioning region level. All analyses were conducted in Stata software version 18.

## Results

The analysis sample for the present study comprises 535068 observations (sample derivation provided in Additional File 6). Table 1 presents the baseline characteristics of our analysis sample. We observe a long-term mental health problem gap of 36·4% (90·1–53·7) in labour force participation in the analysis sample. The sample average NHSTT supply of appointments per referral was 4·45 (1·03 SD). The proportion of observations that were female, single, claimed benefits, had long-term physical health problems, had lower education qualifications, and lived in more deprived areas was more common in the long-term mental health problem sub-sample. To note, in this sub-sample, 60·8% (31153) claimed benefits, and 61·4% (31448) had a long-term physical health problem; more than double the proportions observed in the comparator group.

**Table 1:** Baseline characteristics

	Long-term mental health problem not reported (*N* = 483 861)	Long-term mental health problem reported (*N* = 51 207)	Analysis sample (*N* = 535 068)
***Outcomes:***			
Labour force participation			
* Out of the labour force*	48 068 (9·9%)	23 718 (46·3%)	71 786 (13·4%)
* Labour force participant*	435 793 (90·1%)	27 489 (53·7%)	463 282 (86·6%)
***Exposure (NHSTT supply measure)***:			
Three-month average total appointments per referrals	4·451 ± 1·029	4·433 ± 1·001	4·449 ± 1·026
***Controls***:			
Three-month average waiting time per region	15·895 ± 12·071	16·201 ± 12·037	15·924 ± 12·068
Sex of respondent			
* Male*	236 862 (49·0%)	19 716 (38·5%)	256 578 (48·0%)
* Female*	246 999 (51·0%)	31 491 (61·5%)	278 490 (52·0%)
Marital status: single			
* Married or in civil partnership*	269 322 (55·7%)	19 595 (38·3%)	288 917 (54·0%)
* Single*	214 539 (44·3%)	31 612 (61·7%)	246 151 (46·0%)
Claimed state benefits or tax credits			
* Does not claim benefits*	356 387 (73·7%)	20 054 (39·2%)	376 441 (70·4%)
* Benefits claimant*	127 474 (26·3%)	31 153 (60·8%)	158 627 (29·6%)
Long-term physical health problem			
* None reported*	376 130 (77·7%)	19 759 (38·6%)	395 889 (74·0%)
* Long-term physical health problem reported*	107 731 (22·3%)	31 448 (61·4%)	139 179 (26·0%)
Age (banded)			
* 18–24*	44 980 (9·3%)	4 646 (9·1%)	49 626 (9·3%)
* 25–29*	45 923 (9·5%)	4 536 (8·9%)	50 459 (9·4%)
* 30–34*	55 528 (11·5%)	5 010 (9·8%)	60 538 (11·3%)
* 35–39*	58 446 (12·1%)	5 370 (10·5%)	63 816 (11·9%)
* 40–44*	56 147 (11·6%)	5 672 (11·1%)	61 819 (11·6%)
* 45–49*	60 897 (12·6%)	6 560 (12·8%)	67 457 (12·6%)
* 50–54*	61 462 (12·7%)	7 089 (13·8%)	68 551 (12·8%)
* 55–59*	55 275 (11·4%)	6 797 (13·3%)	62 072 (11·6%)
* 60–65*	45 203 (9·3%)	5 527 (10·8%)	50 730 (9·5%)
Highest level of qualification			
* No qualifications*	34 180 (7·1%)	8 327 (16·3%)	42 507 (7·9%)
* Other qualifications*	35 582 (7·4%)	2 845 (5·6%)	38 427 (7·2%)
* Below NQF Level 2*	51 486 (10·6%)	8 064 (15·7%)	59 550 (11·1%)
* NQF level 2*	72 057 (14·9%)	8 994 (17·6%)	81 051 (15·1%)
* Trade apprenticeships*	15 949 (3·3%)	1 513 (3·0%)	17 462 (3·3%)
* NQF level 3*	80 841 (16·7%)	8 098 (15·8%)	88 939 (16·6%)
* NQF level 4 and above*	193 766 (40·0%)	13 366 (26·1%)	207 132 (38·7%)
Number of dependent children in household			
* None*	263 277 (54·4%)	33 293 (65·0%)	296 570 (55·4%)
* 1*	92 992 (19·2%)	8 076 (15·8%)	101 068 (18·9%)
* 2*	90 793 (18·8%)	6 482 (12·7%)	97 275 (18·2%)
* 3*	26 932 (5·6%)	2 282 (4·5%)	29 214 (5·5%)
* 4+*	9 867 (2·0%)	1 074 (2·1%)	10 941 (2·0%)
Index of multiple deprivation (IMD) decile			
* 1 (Most deprived)*	52 464 (10·8%)	10 304 (20·1%)	62 768 (11·7%)
* 2*	50 836 (10·5%)	7 454 (14·6%)	58 290 (10·9%)
* 3*	49 579 (10·2%)	6 358 (12·4%)	55 937 (10·5%)
* 4*	48 564 (10·0%)	5 423 (10·6%)	53 987 (10·1%)
* 5*	48 139 (9·9%)	4 559 (8·9%)	52 698 (9·8%)
* 6*	46 022 (9·5%)	4 022 (7·9%)	50 044 (9·4%)
* 7*	48 397 (10·0%)	3 826 (7·5%)	52 223 (9·8%)
* 8*	46 887 (9·7%)	3 465 (6·8%)	50 352 (9·4%)
* 9*	45 981 (9·5%)	3 015 (5·9%)	48 996 (9·2%)
* 10 (Least deprived)*	46 992 (9·7%)	2 781 (5·4%)	49 773 (9·3%)
Financial year (April-March)			
* 2015m4-2016m3*	104 826 (21·7%)	9 843 (19·2%)	114 669 (21·4%)
* 2016m4-2017m3*	100 289 (20·7%)	9 927 (19·4%)	110 216 (20·6%)
* 2017m4-2018m3*	98 839 (20·4%)	10 380 (20·3%)	109 219 (20·4%)
* 2018m4-2019m3*	93 486 (19·3%)	10 521 (20·5%)	104 007 (19·4%)
* 2019m4-2020m3*	86 421 (17·9%)	10 536 (20·6%)	96 957 (18·1%)
Quarter			
* April-June*	121 643 (25·1%)	12 490 (24·4%)	134 133 (25·1%)
* July-September*	126 603 (26·2%)	13 461 (26·3%)	140 064 (26·2%)
* October-December*	122 251 (25·3%)	12 933 (25·3%)	135 184 (25·3%)
* January-March*	113 364 (23·4%)	12 323 (24·1%)	125 687 (23·5%)

Statistics presented include the mean and standard deviation for continuous measures or counts and proportions (represented as percentages) for categorical and binary measures used in our model specification. From left to right, the columns present statistics for the participants who did not report a long-term mental health problem, the participants who reported a long-term mental health problem, and the combined analysis sample

Table 2 presents the baseline proportions of our outcome when grouping APS participants by whether they lived in regions with low or high NHSTT supply. Comparing low and high supply commissioning regions, we observe a smaller proportion of individuals who report a mental health problem (-0·7%) and a higher proportion of labour force participation in regions with high NHSTT supply. The difference in average NHSTT supply for the sub-samples is 3·06 appointments per referral received.

**Table 2:** Baseline outcome, treatment status, and NHSTT supply measure by commissioning region activity

	Low supply commissioning region	High supply commissioning region	Combined sample
	(*N* = 89 036)	(*N* = 85 565)	(*N* = 174 601)
***Outcomes:***			
Labour force participation			
* Out of the labour force*	13 347 (15·0%)	10 473 (12·2%)	23 820 (13·6%)
* Labour force participant*	75 689 (85·0%)	75 092 (87·8%)	150 781 (86·4%)
***Mental health problem indicator***			
Long-term mental health problem			
* None-reported*	80 409 (90·3%)	77 890 (91·0%)	158 299 (90·7%)
* Reported*	8 627 (9·7%)	7 675 (9·0%)	16 302 (9·3%)
***Exposure (NHSTT supply measure)***:			
Three-month average total appointments per referrals	2·944 ± 0·364	6·008 ± 0·557	4·446 ± 1·602

Statistics presented include the mean and standard deviation for continuous measures or counts and proportions (represented as percentages) for categorical and binary measures used in our model specification. From left to right, the columns present statistics for the participants who lived in a commissioning region with low NHSTT supply compared to the sample average, participants who lived in a commissioning region with high NHSTT supply compared to the sample average, and the combined sub-samples

The main result of our study is presented in Table 3, reflecting the association between the regional supply of NHSTT and the probability of labour force participation. We find that NHSTT supply is positively associated with the probability of labour force participation across reporting of long-term mental health problems, and no significant association with the sample outcome gap in the unadjusted model (column (1); Table 3). When adjusting for covariates and fixed effects (column (2); Table 3), the marginal effect of NHSTT supply on labour force participation drops out for individuals who did not report a long-term mental health problem, and the association with the outcome gap is now statistically significant. Holding all else equal, a one appointment increase in the regional NHSTT supply measure is associated with a 1·08 percentage point increase in the probability of labour force participation for individuals who reported a long-term mental health problem, and a 0·92 percentage point reduction in the labour force participation gap observed in the analysis sample.

**Table 3:** The association between the supply of NHS talking therapies and labour force participation

	(1)		(2)	
	Labour force participation (unadjusted)		Labour force participation (adjusted)	
	Coefficient	95% C.I.	Coefficient	95% C.I.
***Indicator of long-term MHP***:				
$\:I\left\{{MHP}_{i,t}=1\right\}$	-0.4066^***^	(-0.4484, -0.3648)	-0·2613^***^	(-0·2954, -0·2273)
***Long-term mental health problem not reported***:				
Appointments per referrals	0·00593^**^	(0·002, 0·0099)	0·0017	(-0·0027, 0·006)
***Long-term mental health problem reported***:				
Appointments per referrals	0·0156^*^	(0·0033, 0·0279)	0·0108^**^	(0·00314, 0·0185)
***Relative association with outcome gap***:				
Appointments per referrals	0·00967	(-0·0002, 0·0196)	0·0092^*^	(0·0018, 0·0165)
***Covariates***:				
Waiting time			0	(-0·0003, 0·0004)
$\:I\left\{{MHP}_{i,t}=1\right\}\:$X waiting time			0	(-0·0005, 0·0005)
Biological sex: female			-0·0424^***^	(-0·0483, -0·0366)
Marital status: single			0·011^***^	(0·0078, 0·0142)
Benefits			-0·2165^***^	(-0·2237, -0·2093)
Long-term physical health problem			-0·0836^***^	(-0·0869, -0·0803)
**Age banded** (base: 18–24)				
25–29			0·0102^***^	(0·0061, 0·0143)
30–34			0·0207^***^	(0·0158, 0·0257)
35–39			0·0334^***^	(0·0285, 0·0384)
40–44			0·0394^***^	(0·0347, 0·0441)
45–49			0·0351^***^	(0·0307, 0·0396)
50–54			0·0137^***^	(0·0095, 0·0178)
55–59			-0·017^***^	(-0·0217, -0·0123)
60–65			-0·065^***^	(-0·071, -0·059)
**Highest level of qualification** (base: none)				
Other qualifications			0·1595^***^	(0·1529, 0·1661)
Below NQF Level 2			0·1546^***^	(0·1483, 0·1608)
NQF level 2			0·1944^***^	(0·1873, 0·2016)
Trade apprenticeships			0·191^***^	(0·1842, 0·1979)
NQF level 3			0·2202^***^	(0·2132, 0·2271)
NQF level 4 and above			0·2272^***^	(0·22, 0·2345)
**Dependent children in household** (base: none)				
1			0·0429^***^	(0·0384, 0·0474)
2			0·0242^***^	(0·0191, 0·0294)
3			-0·0283^***^	(-0·0345, -0·0221)
4+			-0·1036^***^	(-0·114, -0·0932)
**IMD decile** (base: 1 (most deprived))				
2			0·0314^***^	(0·0261, 0·0367)
3			0·0481^***^	(0·0431, 0·053)
4			0·053^***^	(0·0478, 0·0583)
5			0·0628^***^	(0·0574, 0·0681)
6			0·0645^***^	(0·0593, 0·0698)
7			0·0634^***^	(0·058, 0·0687)
8			0·0649^***^	(0·0594, 0·0704)
9			0·0615^***^	(0·0553, 0·0677)
10 (least deprived)			0·0544^***^	(0·0484, 0·0604)
Constant			0·7255^***^	(0·7032, 0·7478)
**Specification**:				
Labour force participation outcome	Yes		Yes	
Regional and individual-level covariates	No		Yes	
Interacted region and time period fixed effects	No		Yes	
Mean ± SD of supply measure	4·449 ± 1·026			
Sample average of outcome (%)	86·58 (*N* = 463282)			
Observations (n)	535068		535068	

Coefficients presented with 95% C.I.s in parentheses. Region Fixed Effect: commissioning region of residence. Time Period Fixed Effect: financial year and quarter when survey was completed. Standard errors are cluster robust at the commissioning region level, and estimated with C.I.s using delta method. Stars indicate statistical significance as follows: *p < 0·05, **p < 0·01, ***p < 0·001

The remaining additional analyses used to test our main findings are presented in Figure S1 in Additional File 7, and Tables 4, 5 and 6. We find that variability in the NHSTT supply measure was uncorrelated with covariates introduced in the adjusted model specifications (Figure S1 in Additional File 7).

Table 4 presents the results of our financial year sample stratification to assess the consistency of our main result over the study period. We find a statistically significant association between the regional supply of NHSTT and the probability of labour force participation for APS participants who reported a long-term mental health problem for all financial year subsamples except April 2019 to March 2020 (column (5); Table 4). From this subsample analysis, we find the main result of our study, the association between the supply of NHSTT and the observed gap in labour force participation attributable to long-term mental health problems, was driven by participants who completed the survey in the 2015/16, 2016/17, and 2018/19 financial years (columns (1), (2), and (4); Table 4).

**Table 4:** Assessing the consistency of the main result during the study period

Average marginal effect of appointments per referral	(1)	(2)	(3)	(4)	(5)
	2015/16	2016/17	2017/18	2018/19	2019/20
Long-term mental health problem not reported	0.0004 (-0.0063, 0.0071	0.0028 (-0.0072, 0.0128)	0.0074 (-0.0021, 0.0168)	0.0036 (-0.005, 0.0121)	-0.0051 (-0.0149, 0.0047)
Long-term mental health problem reported	0.0137* (0.0025, 0.0249)	0.0158* (0.0014, 0.0302)	0.0170* (0.0026, 0.0315)	0.0185** (0.0048, 0.0322)	0.0005 (-0.0136, 0.0146)
Relative association of NHSTT supply with outcome gap	0.0133* (0.0026, 0.0239)	0.0130* (0.0015, 0.0245)	0.0097 (-0.0008, 0.0201)	0.015* (0.003, 0.0269)	0.0056 (-0.0054, 0.0165)
**Specification**:					
Labour force participation outcome	Yes	Yes	Yes	Yes	Yes
Regional and individual-level covariates	Yes	Yes	Yes	Yes	Yes
Interacted region and quarter fixed effects	Yes	Yes	Yes	Yes	Yes
Mean ± SD of exposure	4.494 ± 1.121	4.601 ± 1.044	4.457 ± 0.985	4.371 ± 0.965	4.299 ± 0.969
Baseline % of outcome	86.16 (*N* = 98799)	86.23 (*N* = 95039)	86.61 (*N* = 94595)	86.80 (*N* = 90278)	87.23 (*N* = 84576)
Observations (n)	114669	110216	109219	104007	96957

Coefficients presented with 95% C.I.s in parentheses. Regional-Level Covariate: leading three-month average of NHS Talking Therapies waiting time to enter treatment from referral per commissioning region. Individual-Level Covariates: biological sex: female, marital status: single, benefits claimant, self-reported long-term physical health problem, age (banded), highest level of attained qualification, number of dependent children living in household under the age of 19, IMD decile associated with residence. Region Fixed Effect: commissioning region of residence. Time Period Fixed Effect: quarter when survey was completed. Standard errors are cluster robust at the commissioning region level, and estimated with C.I.s using delta method. Stars indicate statistical significance as follows: *p < 0.05, **p < 0.01, ***p < 0.001

In Table 5 we present the estimated results when using the alternative model outcomes, non-repeated APS participants, more restrictive mental health problem indicators, and expanding the interaction term to include long-term physical health problem covariate. When using the paid work outcome (column (1); Table 5), compared to our main results, we find smaller (-0.2 percentage points) but statistically significant associations between being in paid work and the regional supply of NHSTT for individuals who report a long-term mental health problem and in the paid work gap observed in the analysis sample. Columns (2) and (3) of Table 5 present the results of our falsification tests; we find no association between the NHSTT supply of appointments per referral and the gap in probability of labour force participation attributed to long-term mental health problems or the probability of reporting religious beliefs. Columns (4) to (6) of Table 5 show that the main results were robust to potential confounding from repeated participants in the sample and were not sensitive to the use of more restrictive definitions of mental health problems. When interacting the long-term physical health covariate with our exposure and mental health problem indicator (column (7); Table 5), we find a consistent albeit marginally smaller (0·1 − 0·2 percentage points) magnitude of effect for our coefficient of interest.

Table 6 presents the results of our sample benefit claim, age, and gender sample stratifications. Across the three sample stratifications checked, we only find significant associations between the NHSTT supply and labour force participation in the sub-samples representing those who did not claim benefits (column (1); Table 6), the 45–65 age group (column (4); Table 6), and male gender (column (5); Table 6).

**Table 5:** Additional analyses results

Average marginal effect of appointments per referral	(1)	(2)	(3)	(4)	(5)	(6)	(7)
	Paid work outcome	Falsification test: excluded economic inactivity categories	Falsification test: religious beliefs	Non-repeated participants	Main health problem was mental health	Work-limiting mental health problem	Physical health problem included in exposure interaction
Long-term mental health problem not reported	0.001 (-0.0033, 0.0053)	0.0024 (-0.0014, 0.0061)	0.002 (-0.0052, 0.0093)	0·0021 (-0·0028, 0·0069)	0·0021 (-0·0021, 0·0064)	0·002 (-0·0022, 0·0063)	0·0018 (-0·0025, 0·0061)
Long-term mental health problem reported	0.0077* (0.0004, 0.0151)	0.0012 (-0.0046, 0.007)	0.0064 (-0.0021, 0.0149)	0·0117** (0·0034, 0·0199)	0·0106* (0·0021, 0·019)	0·011* (0·0016, 0·0204)	0·0096** (0·0028, 0·0165)
Relative association with outcome gap	0.0068* (0.0001, 0.0134)	-0.0012 (-0.0059, 0.0035)	0.0044 (-0.0007–0.0095)	0·0096* (0·0019, 0·0174)	0·0084* (0·0005, 0·0163)	0·009* (0·0002, 0·0177)	0·0078* (0·0016, 0·0141)
**Specification**							
Outcome	Whether APS participant reported being in paid work	Labour force participation using excluded economic inactivity categories	Whether APS participant reported religious beliefs.	Labour force participation	Labour force participation	Labour force participation	Labour force participation
Regional and individual-level covariates	Yes	Yes	Yes	Yes	Yes	Yes	Yes
Interacted region and time period fixed effects	Yes	Yes	Yes	Yes	Yes	Yes	Yes
Mean ± SD of supply measure	4.449 ± 1.026	4.455 ± 1.024	4.448 ± 1.026	4·454 ± 1·035	4·45 ± 1·026	4·45 ± 1·026	4·449 ± 1·026
Sample average of outcome (%)	82.90 (*N* = 442717)	88.87 (*N* = 463301)	61.93 (*N* = 366618)	86·54 (*N* = 332451)	86·67 (*N* = 462374)	86·67 (*N* = 462374)	86·58 (*N* = 463282)
Observations (n)	534037	521324	591977	384159	533488	533488	535068

Coefficients presented with 95% C.I.s in parentheses. Regional-Level Covariate: leading three-month average of NHS Talking Therapies waiting time to enter treatment from referral per commissioning region. Individual-Level Covariates: biological sex: female, marital status: single, benefits claimant, self-reported long-term physical health problem, age (banded), highest level of attained qualification, number of dependent children living in household under the age of 19, IMD decile associated with residence. Region Fixed Effect: commissioning region of residence. Time Period Fixed Effect: quarter when survey was completed. Standard errors are cluster robust at the commissioning region level, and estimated with C.I.s using delta method. Stars indicate statistical significance as follows: *p < 0.05, **p < 0.01, ***p < 0.001

**Table 6:** Benefit claims, age, and gender sample stratification results

Average marginal effect of appointments per referral	(1)	(2)	(3)	(4)	(5)	(6)
	Did not report benefits	Reported benefits	Aged 18–44	Aged 45–65	Male participants	Female participants
Long-term mental health problem not reported:	0·001 (-0·0023, 0·0043)	0·0056 (-0·0048, 0·0159)	0·0011 (-0·0046, 0·0067)	0·0021 (-0·004, 0·0081)	-0·0001 (-0·0045, 0·0042)	0·004 (-0·0024, 0·0104)
Long-term mental health problem reported:	0·0089** (0·0023, 0·0154)	0·0077 (-0·0036, 0·019)	0·0075 (-0·0021, 0·017)	0·0139** (0·005, 0·0229)	0·0139** (0·0053, 0·0225)	0·0086 (-0·0004, 0·0177)
Relative association with outcome gap:	0·0079* (0·0016, 0·0142)	0·0021 (-0·0047, 0·009)	0·0064 (-0·0022, 0·0151)	0·0119** (0·004, 0·0197)	0·014** (0·0052, 0·0228)	0·0047 (-0·0027, 0·012)
**Specification**						
Labour force participation outcome	Yes	Yes	Yes	Yes	Yes	Yes
Regional and individual-level covariates	Yes	Yes	Yes	Yes	Yes	Yes
Interacted region and time period fixed effects	Yes	Yes	Yes	Yes	Yes	Yes
Mean ± SD of supply measure	4·466 ± 1·026	4·41 ± 1·027	4·44 ± 1·035	4·46 ± 1·015	4·45 ± 1·026	4·448 ± 1·027
Sample average of outcome (%)	95 *N* = 357618	66·62 *N* = 105676	88·4 *N* = 253052	84·5 *N* = 210243	91·72 *N* = 235334	81·85 *N* = 217241
Observations (n)	376440	158624	286257	248808	256578	278490

Coefficients presented with 95% C.I.s in parentheses. Regional-Level Covariate: leading three-month average of NHS Talking Therapies waiting time to enter treatment from referral per commissioning region. Individual-Level Covariates: biological sex: female (not included in columns (5) and (6)), marital status: single, benefits claimant (not included in columns (1) and (2)), self-reported long-term physical health problem, age (banded) (not included in columns (3) and (4)), highest level of attained qualification, number of dependent children living in household under the age of 19, IMD decile associated with residence. Region Fixed Effect: commissioning region of residence. Time Period Fixed Effect: quarter when survey was completed. Standard errors are cluster robust at the commissioning region level, and estimated with C.I.s using delta method. Stars indicate statistical significance as follows: *p < 0.05, **p < 0.01, ***p < 0.001

## Discussion

We examined the relationship between the regional supply of NHSTT and labour force participation for those with and without a long-term mental health problem by pooling national cross-sectional survey data and administrative reports on NHSTT activity.

When stratifying the analysis sample by commissioning regions categorised as low or high NHSTT supply to estimate raw sample statistics, we observed a 0·7% lower proportion of observations with a long-term mental health problem and a 2·8% higher proportion of labour force participation in regions of high supply. These statistics could suggest that, despite the claim that funding is allocated based on predicted need, high-supply regions had healthier populations and higher levels of labour force participation to begin with. An alternative interpretation is that high-supply regions allocated more funding to mental health services because they served populations with greater need, which, in turn, resulted in better health and labour market outcomes. However, we utilise interacted commissioning region and time period fixed effects in our statistical approach to circumvent these potential sources of bias. Provided that the need for mental health treatment within a commissioning region evolves smoothly across our measure of seasonality, our results control for baseline differences and underlying need profiles, isolating only the deviations for both NHSTT supply and labour force participation.

Our main finding was a statistically and economically significant positive association between a one appointment increase in the regional supply of NHSTT appointments offered per referral received and the probability of labour force participation for individuals with a long-term mental health problem. Moreover, this association translates to a 0·92 percentage point reduction in the labour force participation gap between those with and without a long-term mental health problem in the APS sample.

The results of our observational study are consistent with improvements in productivity and employment prospects associated with access to psychological therapy interventions in low- and middle-income countries, as found in randomised controlled trials [3, 5]. Additionally, our main finding and results obtained when using the additional paid work outcome complement clinical impact studies of healthcare systems in high-income countries that use waitlist and event study designs and report small, short- and long-term gains in employment and earnings associated with completing NHSTT treatment [18, 19]. However, to the best of our knowledge, the present study is the first to estimate the association between the regional supply of NHSTT and an individual-level labour force participation outcome constructed using the International Labor Organization definition of economic activity. Traditional employment and productivity outcomes often consider individuals who are unemployed and individuals who are unavailable and not searching for work as the same. These groups can reflect vastly different perspectives on the labour market and aggregated societal impacts. By choosing a labour force participation outcome, we have demonstrated that the regional supply of NHSTT might relate to the decision of an individual to declare themselves available for work using a sample representative of local populations – not just those who interacted with the NHSTT service – providing crucial insight into improving the health and livelihood of those affected by mental health problems through policy that improves access to mental healthcare.

Our results are based on an intention-to-treat analysis because we cannot establish whether individuals in the sample accessed and completed an NHSTT treatment. This implies that we cannot rule out that the underlying mechanisms behind our main finding are the direct effects of using NHSTT services. However, there are low rates of NHSTT treatment uptake among individuals with mental health needs [20–24]. Therefore, we favour the hypothesis regarding the wider perspective of non-health spillover from the regional supply of NHSTT on labour force participation. The association we find may reflect indirect effects arising from the NHSTT model objectives of increasing mental health awareness, which we capture through the regional supply measure. For example, it is plausible that NHSTT services were more visible in commissioning regions that offered more appointments per referral received, which could represent improved help-seeking behaviour, destigmatisation of mental health problems, and changing referral and management behaviours of GPs in primary care networks.

We did not find an association between the supply of NHSTT and the probability of labour force participation when using the economic activity categories we assumed represent inelastic labour supply at the extensive margin (retirees, students, and those who did not provide a reason), or between the supply of NHSTT and the probability of reporting religious beliefs in our falsification tests. Together, we interpret these results as suggesting that our main finding was not driven by random variation in the APS data. When assessing whether the association was consistent over the study period, we observed a statistically significant relationship between the probability of labour force participation and the regional supply of NHSTT for individuals who reported a long-term mental health problem, across all but the last financial year sub-samples. The main finding of an association between the regional supply of NHSTT and the labour participation gap attributed to long-term mental health problems was driven by observations recorded during the 2015/16, 2016/17, and 2018/19 financial years. A possible cause of this could relate to the decline in respondents to the UK Labour Force Survey, which forms the APS data. From 2015/16 to 2019/20, the available sample decreased by 15% (*N* = 17712), with the largest year on year decrease (*N* = 7050) observed between 2018/19 and 2019/20.

Stratifications of the sample by benefit claims, age group, and gender revealed that our main finding was driven by subgroups of individuals who did not report supplementing their income with benefits, were aged 45–65, or were male. These findings contrast previous studies on the utilisation of mental health treatments; regarding age and gender, previous studies have found that the likelihood of utilising psychotherapies is lower in men compared to women [47, 48], and decreases with age [49]. Although it is worth noting that the effect of completing psychological therapy on earnings in long-term follow-up has been found to be stronger, or driven by, male patients and those aged 25–44 [19, 46]. The difference in target populations of our study could be the cause of the contrasting findings, as our intention-to-treat study assessed the relationship between differences in the supply of NHSTT services, representing accessibility to psychotherapy interventions, and the gaps in labour force participation attributed to mental health problems. Future research incorporating stakeholder involvement should examine these differences to identify potential moderating factors in the relationship between the supply of psychological therapy interventions and labour force participation.

The main strength of our study is that, when using a real-world sample and regional supply measures of NHSTT, we observe how the granular treatment effects previously found potentially translate into indirect effects on labour market outcomes at the population level, which hold under several robustness checks. Additionally, the indicator of having a long-term mental health problem we used captures labour market disparities in line with the global estimates (38%) [50]. When stratifying the sample by this indicator, we observe baseline characteristics aligned with the predictors of mental health problems and a difference in the adjusted measure of labour force participation of 36·4 percentage points, capturing the well-established inequitable labour market prospects faced by individuals who report a long-term mental health problem [15, 51–54].

Limitations of our study relate to potential sources of bias in the APS data. We utilised the available participant information to control for factors related to labour force participation and the demand and supply of NHSTT, as well as interacted commissioning region and time period fixed effects. However, we were neither able to identify individual-level NHSTT use, nor to follow individuals over time to control for unobserved factors traditionally captured by individual-level fixed effects. Study power calculations were not completed because the sample size used in this study is fixed due to data availability. Nonetheless, the large number of observations and regions in the sample allows the detection of economically significant associations with high statistical precision. We cluster our standard errors at the commissioning region level to ensure the analysis can detect small differences in the probability of labour force participation between those who reported and those who did not report a long-term mental health problem. The estimated association between NHSTT supply and the probability of labour force participation gap is larger than the margin of error (half-width) implied by the 95% confidence interval, which would rule out any effect smaller than 0.733 percentage points.

The supply measure we use may be vulnerable to factors that could affect the demand and use of NHSTT service. A highly available NHSTT service may not induce help-seeking behaviour or GP referrals to therapy treatment if the quality of appointments and care interactions is poor, dissuading individuals from seeking NHSTT services. Additionally, patient dropouts linked to low symptom severity or unsuitable referrals could lead to understated supply activity in a region by inflating the denominator of the supply measure. However, given the low standard deviation of the appointments per referral measure in the analysis sample, we argue that commissioning regions must be affected similarly by these factors or that the factors do significantly change the activity of NHSTT services.

A further limitation is that analysis of labour force participation conditional on long-term health problems using survey data is vulnerable to the *justification hypothesis* [53]. The *justification hypothesis* suggests that self-reported mental health problems could be underreported by employed individuals and over-reported by individuals who did not participate in the labour force. We cannot ascertain whether the number of individuals who reported a mental health problem was over- or under-reported. The APS data is designed to capture a nationally representative sample to provide an anonymous ‘snapshot’ of the UK population. The representativeness of the APS was one of the primary reasons we chose to use the data, given these limitations. We aimed to complement the existing literature on the treatment effects of psychological therapy interventions on labour market outcomes in controlled environments and patient populations.

## Conclusion

Combined with previous evidence on the NHSTT model, and similar models around the world, we have demonstrated the potential of an innovative policy that improves access to psychological therapy interventions to be related to the labour force participation of those with long-term mental health problems in local populations. The use of healthcare policy should consider approaches to include these indirect effects when estimating costs and benefits of increasing the supply of interventions, and when commissioning healthcare services based on local population needs.

Future research can build upon these findings by exploring target populations where the expansion of access to mental healthcare interventions can yield efficient and cost-effective improvements in labour force participation. For example, the UK’s benefit scheme is undergoing changes aimed at reducing welfare payments. Our subgroup analyses suggest that the regional supply of NHSTT did not relate to job-searching behaviour among individuals claiming benefits. If these individuals are no longer able to supplement their income through benefits claims, income poverty and economic stress could cause the demand for mental healthcare interventions to increase. An evaluation of how the supply of NHSTT could moderate any systemic poverty created or exacerbated by the benefit system reform would provide crucial insight into the societal management of mental health problems.

## Supplementary Information

Below is the link to the electronic supplementary material.

Supplementary Material 1.

Supplementary Material 2.

Supplementary Material 3.

Supplementary Material 4.

Supplementary Material 5.

Supplementary Material 6.

Supplementary Material 7.

## Data Availability

The dataset created from individual-level data from the Annual Population Survey is not publicly available and was obtained from the UK Data Service via a Secure Access Data Sharing Agreement. Access to this data is restricted to accredited researchers on projects approved by the UK Data Service and provided through a secure virtual environment due to the sensitive information it includes. Information regarding the study and questionnaires can be found at: [https://beta.ukdataservice.ac.uk/datacatalogue/studies/study?id=6721#!/documentation] The publicly available NHS Talking Therapies activity reports used to measure regional supply of psychological therapy interventions, and their supporting information, can be found at: [https://digital.nhs.uk/data-and-information/publications/statistical/psychological-therapies-report-on-the-use-of-iapt-services].

## Abbreviations

NHS: English national health service
NHSTT: NHS talking therapies
GP: General practitioner
UK: United Kingdom
APS: Annual population survey
AME: Average marginal effect
SD: Standard deviation
NQF: National qualifications framework
IMD: Index of multiple deprivation
