# Supplementary material for: Availability of psychological therapies and workforce participation of individuals with long-term mental health problems: a retrospective observational study
Source: Int J Ment Health Syst. 2026 Apr 15;20:9. doi: 10.1186/s13033-026-00706-z (PMC13200466; doi:10.1186/s13033-026-00706-z)
Supplement: Supplementary file 1 — Supplementary Material 1. [file 13033_2026_706_MOESM1_ESM.docx]

**Additional File 1**

**Annual Population Survey Sampling and Interview Information**

The Annual Population Survey is a nationwide repeated cross-sectional study aiming to provide up-to-date annual measures of social and socioeconomic factors, representative of local area populations within each of the countries that constitute the UK (England, Scotland, Wales, and Northern Ireland) on a rolling quarterly basis. The following information was sourced from the official user guides produced by the data controllers [1]. From 2006, the APS was formed from two interlinked sources of data. The first is the quarterly Labour Force Survey, a continuous nationwide household survey with a cohort study design. The target population for the survey is any persons aged 16-75 who reside in private households or communal establishments, which extends to students who live away from home during their studies but officially report their residence as their family home. For England, the LFS uses a multi-stage stratified (systematic) random rotational sampling approach to recruit and interview participants. Recruited households (and their inhabitants) form a cohort which are followed for five consecutive waves corresponding to the following quarters of a year: January to March, April to June, July to September, and October to December. Each household is planned to be interviewed five times, with each interview taking place 13 weeks apart, ensuring the last interview is completed a year after the first.

A sampling frame constructed from national postal address datasets was used to recruit the wave one sample of households for each quarter. Only private addresses and communal establishments are included in the sampling frame. The sampling frame is ordered by geographic regions within the UK, allowing the wave one sample in each region to be drawn systematically. The number of addresses selected per quarter from the sampling frame is 18,030 for the UK; 14,282 from England, 858 from Wales, 1580 from Scotland, 1300 from Northern Ireland, and roughly nine National Health Service managed accommodation addresses. For England, Scotland, and Wales, the sampling interval, k, is calculated by dividing the total number of addresses by the selected number of households, corresponding to a 1-in-1586 wave one quarterly sample size. Then, a random starting point is chosen from the subset {1,2,…,k}. The address chosen from the random starting point, and every kth address after that, are ‘marked’ as available to invite to participate. After the marked addresses are compiled, they are compared with a used address database, which includes households that have participated in any government/administrative social survey at some point in the past two years. Collating addresses into postcode sectors, a new systematic sample is then drawn by calculating the sampling interval k for each postcode sector to select only addresses that are not included in the used address database.

Households selected to participate are then allocated to one of the 208 UK Interviewer Areas, which are further split to form two/three quotas per area. There are 318 quotas in England. To complete interviews, each quota is divided into 13 stints, which are randomly allocated to the 13 weeks of each quarter. Once allocated, a quota will be covered by an interviewer in the same corresponding week of each subsequent quarter. Most wave one interviews are completed in person, with follow-up wave interviews conducted over the phone. To ensure that no household is included twice in the Annual Population Survey datasets, only data collected in waves one and five of the quarterly LFS are included each year of the APS. However, if the APS datasets are pooled together and a participant completes both the first and fifth wave quarterly LFS interviews, there will be two observations of the participant spaced a year apart.

The APS has a target number of economically active participants per Unitary Authority/Local Authority District (for definition see page 3 of Additional File 1) in England; 510 persons per Local Authority District and 450 per governing regions in London. Therefore, a second source of data is used to complement the quarterly Labour Force Survey wave one and five samples that form the APS. These are the Local Labour Force Survey (LLFS) for England, Scotland, and Wales. LLFSs capture the same information as the quarterly LFS, but differ slightly in their sampling approach and the frequency of interview waves, as they are designed to boost the APS sample sizes to achieve targets for economically active individuals per area. Interview waves occur annually, rather than quarterly, with households being interviewed as part of the sample for four consecutive years. Between two consecutive years, the interview wave design ensures that three quarters of the sample are completing waves two to four, and the other quarter is replaced with new participants.

Clustered random sampling is used to stratify the LLFS by local regions to ensure the APS sample size achieves the target number of economically active participants per Unitary Authority/Local Authority District. The number of households invited to participate in the LLFS, therefore, depends on the quarterly LFS response rates per area, which are reviewed and updated annually. For some areas, the number of conducted interviews from waves one and five of the quarterly LFS exceeds the target of economically active participants in the previous year, and so, no LLFS boost sample is required. In areas where previous LLFS sample boosts occurred, the combined sample size from the quarterly LFS waves and the LLFS boost are used to predict response rates and attrition between waves. From this, a projected number of achieved interviews per area is estimated for the three following years to ascertain if the combined LFS and LLFS sample will meet the population targets. A tolerance of 10 percent between the projection and the population target is used to decide the adjustment required to the LLFS boost sample. If the projected achieved interviews are within 10 percent of the target, no changes to the boost sample are required in the following year. Should the projected number fall outside the 10% tolerance rate of the target, the boost sample is increased or decreased in the following year to re-align the number of interviews with the population target at the end of the three-year projection.

Once a household is selected for the LFS or LLFS samples, a letter is sent in advance of the wave one interview to provide information on the survey and an estimated date and time of the wave one interviews. BLAISE Computer Assisted Interviewing (CAI) is used to obtain responses to the survey questionnaire. Dependent interviewing techniques are employed during follow-up waves; the interviewer will present the previous response to a question completed in the last interview as part of asking the question in the current wave in order to improve the quality of data collected. These occur predominantly in a set of core questions asked at each wave, designed to comprehensively capture factors related to economic activity. Proxy interviews can be implemented when some household members are unavailable at the time of the interview; the available members provide responses for the unavailable residents. Whilst there is a requirement to provide answers to all questions relating to economic activity for the interview record to be used in the APS dataset, there are some cases where participants can provide “no answer” or “don’t know”. As the questionnaire must be of an appropriate length, not all information can be obtained through direct questions. Derived variables are constructed on the response to several core questions in such cases.

**Local Authority Districts, Unitary Authorities, and Commissioning Regions in England**

In England, regions are governed by either a single (Unitary Authorities, Metropolitan Districts, London Boroughs) or two-tier structure (County and District councils), depending on their size. Unitary Authorities are for larger cities/towns which do not require division into districts within them, and Local Authority Districts are the smaller level regions that constitute County council districts.

For our study period, commissioning regions in England were Clinical Commissioning Groups. Clinical Commissioning Groups were formed in 2012 as part of the Health and Social Care Act, and were replaced in 2022 by Integrated Care Systems [2]. Clinical Commissioning Groups were a group of GP practices in a specified area of England, allocated a budget to commission NHS services for the patient population they served [3]. NHS England would oversee Clinical Commissioning Groups. However, Clinical Commissioning Groups had flexibility to choose among which services they would provide and which they would source externally.

During the study period, Local Authority Districts and Clinical Commissioning Group regions of operation were closely matched. When these commissioning regions were first formed, guidance produced by the UK Government set out that Clinical Commissioning Groups should define clear regional boundaries encompassing the patients for whom they commission services [4]. These boundaries should not cross Unitary or Local Authority Districts without good cause to do so. As a result, most (86%) of the Clinical Commissioning Group regions in England during the study period shared geographical boundaries with Local Authority Districts, with the goal of local government members working together to ensure resources are efficiently used [5].

**References**

1. Office for National Statistics, Social Survey Division, 2025, Annual Population Survey, 2004-2024: Secure Access, 33rd Edition. UK Data Service (SN: 6721). http://doi.org/10.5255/UKDA-SN-6721-32.

2. NHS England. Integrated care systems (ICSs). 2024. https://www.england.nhs.uk/commissioning/who-commissions-nhs-services/ccg-ics/. Accessed 20 May 2024.

3. NHS. Clinical Commissioning Group. 2024. https://www.datadictionary.nhs.uk/nhs_business_definitions/clinical_commissioning_group.html. Accessed 20 May 2024.

4. Department of Health and Social Care. Guidance for Clinical Commissioning Groups September 2011. GOV.UK. 2011. https://www.gov.uk/government/publications/guidance-for-clinical-commissioning-groups-september-2011. Accessed 5 June 2024.

5. Baker C, Sandford M. Mapping the Places. Research Briefing Report No.: 08778. House of Commons; 2020. https://commonslibrary.parliament.uk/research-briefings/cbp-8778/. Accessed 5 June 2024.
