# Supplementary material for: Availability of psychological therapies and workforce participation of individuals with long-term mental health problems: a retrospective observational study
Source: Int J Ment Health Syst. 2026 Apr 15;20:9. doi: 10.1186/s13033-026-00706-z (PMC13200466; doi:10.1186/s13033-026-00706-z)
Supplement: Supplementary file 2 — Supplementary Material 2. [file 13033_2026_706_MOESM2_ESM.docx]

**Additional File 2**

**Table S1: Variables Used to Derive Economic Activity in the Annual Population Survey**

| **APS Variable** | **Description/Survey Question** | **Variable Options** | **Additional Information** |
| --- | --- | --- | --- |
| AGE | Age of respondent | 0-99 |  |
| SCHM12 | ‘Last week were you on any of the following schemes/programmes’ | 1. Work Club or Enterprise Club. 2. New Enterprise Allowance. 3. Work Experience. 4. Work Trial. 5. Work Programme. 6. Training for Success. 7. Steps 2 Success. 8. Training for Work. 9. Get ready for work. 10. Any other government training scheme. 11. Or none of these? 12. Just 16 and non-response this time. | Applies if aged 16-64.  Options 6 and 7 only available if completing the survey in Northern Ireland. Options 8 and 9 only available if completing the survey in Scotland. |
| COUNTRY | Country within UK. | 1. England. 2. Wales. 3. Scotland. 4. Scotland North of Caledonian Canal. 5. Northern Ireland. |  |
| FUND12 | Funding of work schemes | 1. A scheme in England funded by the Skills Funding Agency or the can Funding Agency. 2. A programme in Wales funded by the Welsh Government. 3. A programme in Scotland run by Skills Development Scotland. | Applies to respondents on certain type of training scheme (SCHM12 = 1,5,8,9,50). |
| TYPSCH12 | ‘On Government scheme/programme, were you mainly…’ | 1. Working for an employer. 2. Temporarily away from an employer. 3. Working for a voluntary organisation/charity. 4. Undertaking some other form of voluntary or community work. 5. Working for an environmental taskforce. 6. In full-time or part-time study. 7. Temporarily away from full-time or part-time study. 8. Receiving help setting up as self-employed. 9. On a project providing work experience or practical training. 10. Undertaking some other form of employment training. 11. Or some other situation not listed. 12. Don’t know. | Applies to respondents on Government training schemes (SCHM12 = 1-50). |
| HELPSE12 | Period when receiving help setting up as self-employed. | 1. Prior to starting a business as self-employed or, 2. After setting up a business as self-employed. | Applies to respondents who reported getting help setting up as self-employed (TYPSCH12=8). |
| YTETJB | Whether had paid job in addition to Government scheme (in reference week). | 1. Yes. 2. No. | Applies to respondents on certain Government training schemes (SCHM12 = 4,6,7,9,10,11,97). |
| STATR | Employment status in main job. | 1. Employee. 2. Self-employed. 3. Government scheme. 4. Unpaid family worker. | Derived from employment questions in the survey. |
| WRKING | Whether did paid work in reference week. | 1. Yes. 2. No. | Applies to anyone not on a training scheme aged >16. |
| JBAWAY | Whether temporarily away from paid work. | 1. Yes. 2. No. 3. Waiting to take up new job/business already obtained. | Applies if WRKING = 2. |
| OWNBUS | Whether doing unpaid work for own business. | 1. Yes. 2. No. | Applies if JBAWAY= 2,3. |
| RELBUS | Whether doing unpaid work for a business owned by a relative. | 1. Yes. 2. No. | Applies if JBAWAY= 2,3. |
| LOOK4 | Whether looking for paid work in last four weeks. | 1. Yes. 2. No. | Applies if respondent did unpaid work or had no current job. |
| LKYT4 | Whether looking for a place on a government scheme in the last four weeks. | 1. Yes. 2. No. | Applies if respondent not seeking work (LOOK4=2). |
| START | Whether could start work within the next two weeks. | 1. Yes. 2. No. | Applies if respondent is looking for, starting a new, or wanting a job. |
| WAIT | Whether waiting to take up job. | 1. Yes. 2. No. | Applies if respondent is not seeking work or training scheme. |
| LIKEWK | Whether would like work. | 1. Yes. 2. No | Applies if respondent is not seeking work/training scheme and not waiting to take up a job (WAIT=2). |
| YSTART | Reason could not start work within two weeks. | 1. Must complete education. 2. Cannot leave present job within 2 weeks. 3. Looking after the family/home. 4. Temporarily sick or injured. 5. Long-term sick or disabled. 6. Other reason. | Applies if respondent is not available to start work (START=2). |
| NOLWM | Main reason for not looking for work in last 4 weeks. | 1. Waiting for the results of an application for a job/being assessed by a training agent. 2. Student. 3. Looking after the family/home. 4. Temporarily sick or injured. 5. Long-term sick or disabled. 6. Believe(s) no jobs available. 7. Not yet started looking. 8. Do(does) not need employment. 9. Retired from paid work. 10. Any other reason. | Applies if respondent is not seeking work/training scheme and not waiting to take up a job (WAIT=2). |

**Table S2: Annual Population Survey Economic Activity Variable Categories and their Proportion of the Starting Sample**

| **Economic Activity Variable Category** | **Labour Force Participation Outcome** | **No Reported Mental Health Problem**  **(N=553 178)** | **Reported Mental Health Problem**  **(N=57 097)** | **Combined Sample**  **(N=610 275)** |
| --- | --- | --- | --- | --- |
| Employee | In the labour force (=1) | 367 071 (66·4%) | 21 615 (37·9%) | 388 686 (63·7%) |
| Self-employed | In the labour force (=1) | 62 900 (11·4%) | 3 221 (5·6%) | 66 121 (10·8%) |
| Government employment & training programmes | In the labour force (=1) | 541 (0·1%) | 193 (0·3%) | 734 (0·1%) |
| Unpaid family worker | In the labour force (=1) | 959 (0·2%) | 103 (0·2%) | 1 062 (0·2%) |
| ILO unemployed | In the labour force (=1) | 16 813 (3·0%) | 3 215 (5·6%) | 20 028 (3·3%) |
| Inactive- seeking, unavailable, student | Excluded | -900 (0·2%) | -60 (0·1%) | -960 (0·2%) |
| Inactive- seeking, unavailable, looking after family, home | Out of the labour force (=0) | 834 (0·2%) | 138 (0·2%) | 972 (0·2%) |
| Inactive- seeking, unavailable, temp sick or injured | Out of the labour force (=0) | 134 (0·0%) | 103 (0·2%) | 237 (0·0%) |
| Inactive- seeking, unavailable, long-term sick, disabled | Out of the labour force (=0) | 81 (0·0%) | 137 (0·2%) | 218 (0·0%) |
| Inactive- seeking, unavailable, other reason | Excluded | -820 (0·1%) | -109 (0·2%) | -929 (0·2%) |
| Inactive- seeking, unavailable, no reason given | Excluded | -156 (0·0%) | -50 (0·1%) | -206 (0·0%) |
| Inactive- not seeking, would like, wait res job app | Excluded | -186 (0·0%) | -21 (0·0%) | -207 (0·0%) |
| Inactive- not seeking, would like, student | Excluded | -2 094 (0·4%) | -243 (0·4%) | -2 337 (0·4%) |
| Inactive- not seeking, would like, looking after fam, home | Out of the labour force (=0) | 6 234 (1·1%) | 1 231 (2·2%) | 7 465 (1·2%) |
| Inactive- not seeking, would like, temp sick, injured | Out of the labour force (=0) | 671 (0·1%) | 601 (1·1%) | 1 272 (0·2%) |
| Inactive- not seeking, would like, long term sick, disabled | Out of the labour force (=0) | 3 566 (0·6%) | 5 594 (9·8%) | 9 160 (1·5%) |
| Inactive- not seeking, would like, believes no job avail | Out of the labour force (=0) | 278 (0·1%) | 80 (0·1%) | 358 (0·1%) |
| Inactive- not seeking, would like, not yet looking | Out of the labour force (=0) | 902 (0·2%) | 128 (0·2%) | 1 030 (0·2%) |
| Inactive- not seeking, would like, doesn’t need job | Excluded | -448 (0·1%) | -55 (0·1%) | -503 (0·1%) |
| Inactive- not seeking, would like, retired | Excluded | -609 (0·1%) | -75 (0·1%) | -684 (0·1%) |
| Inactive- not seeking, would like, other reason | Excluded | -1 698 (0·3%) | -312 (0·5%) | -2 010 (0·3%) |
| Inactive- not seeking, would like, no reason given | Excluded | -15 (0·0%) | -<10 (0·0%) | -15 (0·0%) |
| Inactive- not seeking, not like, waiting results of application | Excluded | -168 (0·0%) | -11 (0·0%) | -179 (0·0%) |
| Inactive- not seeking, would not like, student | Excluded | -14 201 (2·6%) | -866 (1·5%) | -15 067 (2·5%) |
| Inactive- not seeking, would not like, looking after family/home | Out of the labour force (=0) | 24 010 (4·3%) | 2 907 (5·1%) | 26 917 (4·4%) |
| Inactive- not seeking, would not like, temp sick/injured | Out of the labour force (=0) | 733 (0·1%) | 579 (1·0%) | 1 312 (0·2%) |
| Inactive- not seeking, would not like, long-term sick/disabled | Out of the labour force (=0) | 11 160 (2·0%) | 12 822 (22·5%) | 23 982 (3·9%) |
| Inactive- not seeking, would not like, believes no job available | Out of the labour force (=0) | 176 (0·0%) | 33 (0·1%) | 209 (0·0%) |
| Inactive- not seeking, would not like, not yet looking | Out of the labour force (=0) | 611 (0·1%) | 79 (0·1%) | 690 (0·1%) |
| Inactive- not seeking, would not like, doesn’t need job | Excluded | -4 045 (0·7%) | -260 (0·5%) | -4 305 (0·7%) |
| Inactive- not seeking, would not like, retired | Excluded | -26 124 (4·7%) | -1 695 (3·0%) | -27 819 (4·6%) |
| Inactive- not seeking, would not like, other reason | Excluded | -2 876 (0·5%) | -450 (0·8%) | -3 326 (0·5%) |
| Inactive- not seeking, would not like, no reason given | Excluded | -1 164 (0·2%) | -111 (0·2%) | -1 275 (0·2%) |
