# Supplementary material for: Availability of psychological therapies and workforce participation of individuals with long-term mental health problems: a retrospective observational study
Source: Int J Ment Health Syst. 2026 Apr 15;20:9. doi: 10.1186/s13033-026-00706-z (PMC13200466; doi:10.1186/s13033-026-00706-z)
Supplement: Supplementary file 3 — Supplementary Material 3. [file 13033_2026_706_MOESM3_ESM.docx]

**Additional File 3**

**NHS Talking Therapies for Anxiety and Depression Background and Data**

The NHS Talking Therapies (NHSTT) service model was launched in 2008 to expand access to psychological therapy interventions for common mental health problems. A unique feature of this programme is that it collects and reports annual and monthly data on the supply and performance of services at the provider and commissioning region levels. These publicly available administrative reports present official statistics on data collected by service providers, including referrals received (type and quantity) and appointments provided (counts, attendance, and average per completed treatment), as well as waiting times and measures of patient recovery after completing treatment.

The rollout of the service represented a substantial increase in the supply of mental healthcare services across England, which recently reported 1·81 million annual referrals, and of those referrals, 1·24 million individuals accessing the service in 2021/2022 [1]. However, NHSTT faces a significant attrition problem, with less than half of the referred patients not completing their course of treatment [2]. Universal health insurance in England covers the use of NHSTT services. The treatments provided are free at the point of use, removing any confounding effects of health insurance on access and labour force participation. Treatment plans are tailored to the patient under the person-centred care philosophy, offering acceptable interventions delivered in person or remotely that meet the needs of patients entering the service. Individuals can be referred to the service through several pathways, including self-referral, improving the accessibility of these treatments.

We use monthly activity data from January 2015 to March 2020. The NHS operates on a financial year reporting schedule (April to March), and this period includes consistently recorded data measures we wish to study from the NHSTT services. We obtained monthly commissioning region-level statistics, starting with 216 commissioning regions in the financial year April 2015 to March 2016.

**References**

1. NHS Digital. Psychological Therapies, Annual report on the use of IAPT services, 2021-22. 2022. https://digital.nhs.uk/data-and-information/publications/statistical/psychological-therapies-annual-reports-on-the-use-of-iapt-services/annual-report-2021-22. Accessed 7 Dec 2023.

2. Bagri S. Does the NHS Talking Therapies service have an attrition problem? QualityWatch. 2024. https://www.nuffieldtrust.org.uk/news-item/does-the-nhs-talking-therapies-service-have-an-attrition-problem. Accessed 9 May 2025.
