# Supplementary material for: Availability of psychological therapies and workforce participation of individuals with long-term mental health problems: a retrospective observational study
Source: Int J Ment Health Syst. 2026 Apr 15;20:9. doi: 10.1186/s13033-026-00706-z (PMC13200466; doi:10.1186/s13033-026-00706-z)
Supplement: Supplementary file 4 — Supplementary Material 4. [file 13033_2026_706_MOESM4_ESM.docx]

**Additional File 4**

**Table S3: Annual Population Survey Health Variables, Related Questions, and Response Options**

| **APS Variable** | **Question** | **Response Options** | **Created measure** |
| --- | --- | --- | --- |
| QHEALTH1 | “How is your health in general; would you say it was…” | 1. Very good. 2. Good. 3. Fair. 4. Bad. 5. Very bad. | None. |
| LNGLST | “Do you have any physical or mental health conditions or illnesses lasting or expecting to last 12 months or more?” | 1. Yes. 2. No. 3. Don’t know (spontaneous only). 4. Refusal (spontaneous only). | All long-term mental health problem indicators. Physical health problem covariate. |
| LIMITK | “Does this health problem affect the kind of paid work that you might do?” | 1. Yes. 2. No. | Sensitivity Analysis: Work-limiting mental health problem indicator. |
| LIMITA | “Does this health problem affect the amount of paid work that you might do?” | 1. Yes. 2. No. | Sensitivity Analysis: Work-limiting mental health problem indicator. |
| HEAL20 | “Do you have… *code all that apply”* | 1. Problems or disabilities (including arthritis or rheumatism) connected with your arms or hands? 2. … legs or feet? 3. … back or neck? 4. Difficulty in seeing (while wearing spectacles or contact lenses)? 5. Difficulty in hearing? 6. A speech impediment? 7. Severe disfigurements, skin conditions, allergies? 8. Chest or breathing problems, asthma, bronchitis? 9. Heart, blood pressure or blood circulation problems? 10. Stomach, liver, kidney or digestive problems? 11. Diabetes? 12. Depression, bad nerves or anxiety? 13. Epilepsy? 14. Severe or specific learning difficulties? 15. Mental illness or suffer from phobias, panics or other nervous disorders? 16. Progressive illness not included elsewhere (eg cancer no included elsewhere, multiple sclerosis, symptomatic HIV, Parkinson’s disease, Muscular Dystrophy)? 17. Other health problems or disabilities? 18. Autism (including autism spectrum condition, Asperger syndrome)? | All long-term mental health problem indicators. Physical health problem covariate. |
| HEALTH20 | [if more than one response provided to HEAL20] “Which of these is your main health problem/disability?” | 1. Problems or disabilities (including arthritis or rheumatism) connected with your arms or hands? 2. … legs or feet? 3. … back or neck? 4. Difficulty in seeing (while wearing spectacles or contact lenses)? 5. Difficulty in hearing? 6. A speech impediment? 7. Severe disfigurements, skin conditions, allergies? 8. Chest or breathing problems, asthma, bronchitis? 9. Heart, blood pressure or blood circulation problems? 10. Stomach, liver, kidney or digestive problems? 11. Diabetes? 12. Depression, bad nerves or anxiety? 13. Epilepsy? 14. Severe or specific learning difficulties? 15. Mental illness or suffer from phobias, panics or other nervous disorders? 16. Progressive illness not included elsewhere (eg cancer no included elsewhere, multiple sclerosis, symptomatic HIV, Parkinson’s disease, Muscular Dystrophy)? 17. Other health problems or disabilities? 18. Autism (including autism spectrum condition, Asperger syndrome)? | Sensitivity Analysis: Main health problem is a mental health problem indicator and work-limiting mental health problem indicator. |
