# Supplementary material for: Availability of psychological therapies and workforce participation of individuals with long-term mental health problems: a retrospective observational study
Source: Int J Ment Health Syst. 2026 Apr 15;20:9. doi: 10.1186/s13033-026-00706-z (PMC13200466; doi:10.1186/s13033-026-00706-z)
Supplement: Supplementary file 5 — Supplementary Material 5. [file 13033_2026_706_MOESM5_ESM.docx]

**Additional File 5**

**Table S4: Variable Descriptions**

| **Variable** | **Description** | **Source** |
| --- | --- | --- |
| **Outcome:** |  |  |
| Labour Force Participation | Binary variable taking a value of one if individual *i* is self-employed, employed (both full and part-time), on government training or employment, or unemployed (ILO definition of unemployment). A value of zero is given to those who are not willing or available for work. | Annual Population Survey |
| **Mental Health Problem Indicators** ($I\left\{ {MHP}_{i,t}=1 \right\}$): |  |  |
| Long-term mental health problem (*main analysis indicator*) | Binary indicator variable taking a value of one if individual *i* reports that they have a long-term (or expected to be long-term) mental health problem, and zero otherwise. | Annual Population Survey |
| Main long-term health problem is a mental health problem | Binary indicator variable taking a value of one if individual *i* reports that they have a long-term (or expected to be long-term) mental health problem, and this is their main health problem if they report more than one, zero otherwise. | Annual Population Survey |
| Work-Limiting Mental Health Problem | Binary indicator variable taking a value of one if individual *i* reports that they have a long-term (or expected to be long-term) mental health problem which affects the kind of work they can do, and this is their main health problem if they report more than one, zero otherwise. | Annual Population Survey |
| **Exposure**: |  |  |
| Regional Supply of NHSTT | The three-month total appointments offered to patients divided by the three-month total referrals received by NHS Talking Therapies services in individual *i*'s commissioning region prior to completing the survey. | NHS Talking Therapies Activity Reports |
| **Covariates** ($\boldsymbol{X}_{i,t}$): |  |  |
| Waiting Time | The three-month average of regional median waiting time from referral to entering treatment of NHS Talking Therapies services prior to completing the survey in individual *i*'s commissioning region. | NHS Talking Therapies Activity Reports |
| Biological Sex: Female | Binary variable taking a value of one if individual *i*'s reported biological sex is female, and zero if male. | Annual Population Survey |
| Age Bands | Age of survey respondent in bands: 18-24, 25-29, 30-34, 35-39, 40-44, 45-49, 50-54, 55-59, 60-65. | Annual Population Survey |
| Marital Status: Single | Binary variable taking a value of one if individual *i*'s marital status is single, divorced, or widowed, and zero otherwise. | Annual Population Survey |
| Number of Dependent Children in Household | Number of dependent children (age<19) living in individual *i*'s household. | Annual Population Survey |
| Highest Level of Qualification | Highest level of qualification attained by survey respondent *i*: no qualifications, other qualifications, below NQF level 2, NQF level 2, trade apprenticeship, NQF level 3, or NQF level 4 and above. | Annual Population Survey |
| Long-Term Physical Health Problem | Binary variable taking a value of one if individual *i* reports a long-term physical health problem, and zero otherwise. | Annual Population Survey |
| Benefits | Binary variable taking a value of 1 if individual *i* reported they claimed at least one state benefit, zero otherwise. | Annual Population Survey |
| Index of Multiple Deprivation (IMD) Decile | IMD ranking (decile) of individual *i*'s Lower Layer Super Output Area of residence. | Annual Population Survey |
| Commissioning Region of Residence Fixed Effect ($\boldsymbol{Region}_{i,r}$) | Binary variable representing the fixed effect for individual *i*’s commissioning region of residence, *r*. | Annual Population Survey |
| Financial Year and Quarter Seasonal Fixed Effect ($\boldsymbol{Period}_{i,t}$) | Binary variable representing the fixed effect for the financial year and quarter *t* of when individual *i* completed the survey. | Annual Population Survey |

**Statistical Analysis – Model Assumptions and Interpretation**

We aim to estimate the unbiased Intention-to-Treat (ITT) association between NHS Talking Therapies supply and the individual-level probability of labour force participation using a linear probability model specification. The ITT association is our chosen target parameter as we investigate the indirect effects of a policy to expand access to evidence-based psychological therapy interventions on all individuals who could benefit from an increased supply of mental healthcare services, not just those who used and completed NHSTT treatment. As such, we interact our NHSTT supply measure with the long-term mental health problem indicator to estimate any association using the full analysis sample. In doing so, the interaction term provides an estimated association between NHSTT supply and any ‘gap’ in labour force participation observed in the sample. This gap reflects the difference in what we assume is the normal workforce participation rate and what we assume is reduced due to the collinear relationship between mental health and labour market outcomes. Notably, the APS does not capture whether participants utilised NHSTT services.

The long-term mental health problem indicator should identify the individuals who were less likely to participate in the labour force and may benefit directly from seeking NHSTT services, or indirectly from more available mental healthcare. This indicator follows the same identification of long-term mental health problems employed in the analysis of English Labour Force Survey data [1–5]. Furthermore, individuals with long-term mental health problems are likely to be in some form of contact with the NHS healthcare system for ongoing management of their condition or to obtain a diagnosis required to receive welfare payments/benefits. This suggests the indicator captures individuals with the necessary mental health literacy to be aware of their symptoms and potentially more knowledgeable about treatment pathways in their area of residence.

To identify the ITT, our key assumption is conditional mean independence. We assume that, conditional on observed covariates, there are no remaining unobserved factors that simultaneously influence both the NHSTT supply measure and the labour force participation outcome [6]. We build the model specification to include fixed effects and covariates, which plausibly isolate the relationship between the NHSTT supply measure and the individual-level probability of labour force participation. Using individuals who did not report a long-term mental health problem as a comparator group in our analysis introduces further assumptions. Given that the APS intends to provide a nationally representative sample for a given quarter of a year, we assume the labour force participation rate of individuals who did not report a long-term mental health problem (excluding students, retirees, and those who do not need paid work) reflects the norm in the English labour market. We also assume these individuals can remain in the workforce without seeking psychological therapy interventions, and their engagement with the service is less likely to be determined by the regional supply of NHSTT.

**References**

1. McDaid D, Park A-L, Davidson G, John A, Knifton L, McDaid S, et al. The economic case for investing in the prevention of mental health conditions in the UK (Summary). London School of Economics and Political Science; 2022. https://www.mentalhealth.org.uk/explore-mental-health/publications/economic-case-investing-prevention-mental-health-conditions-UK. Accessed 11 Jul 2024.

2. Cardoso F, McHayle Z. The economic and social costs of mental ill health. Centre for Mental Health; 2024. https://www.centreformentalhealth.org.uk/publications/the-economic-and-social-costs-of-mental-ill-health/. Accessed 11 Jul 2024.

3. Office for National Statistics. Health, demographic and labour market influences on economic inactivity, UK: 2019 to 2022. 2023. https://www.ons.gov.uk/employmentandlabourmarket/peoplenotinwork/economicinactivity/articles/healthdemographicandlabourmarketinfluencesoneconomicinactivityuk2019to2022/2023-05-19. Accessed 23 Oct 2024.

4. Office for National Statistics. Rising ill-health and economic inactivity because of long-term sickness, UK: 2019 to 2023. 2023. https://www.ons.gov.uk/employmentandlabourmarket/peoplenotinwork/economicinactivity/articles/risingillhealthandeconomicinactivitybecauseoflongtermsicknessuk/2019to2023. Accessed 23 Oct 2024

5. Atwell S, Vriend M, Rocks C, Finch D, Farrington-Douglas J. What we know about the UK’s working-age health challenge. The Health Foundation. 2023. https://www.health.org.uk/publications/long-reads/what-we-know-about-the-uk-s-working-age-health-challenge. Accessed 23 Oct 2024.

6. Imbens GW, Wooldridge JM. Recent Developments in the Econometrics of Program Evaluation. Journal of Economic Literature. 2009;47:5–86. https://doi.org/10.1257/jel.47.1.5.
