# Supplementary material for: Availability of psychological therapies and workforce participation of individuals with long-term mental health problems: a retrospective observational study
Source: Int J Ment Health Syst. 2026 Apr 15;20:9. doi: 10.1186/s13033-026-00706-z (PMC13200466; doi:10.1186/s13033-026-00706-z)
Supplement: Supplementary file 6 — Supplementary Material 6. [file 13033_2026_706_MOESM6_ESM.docx]

**Additional File 6**

**Table S5: Analysis Sample Derivation**

|  | **Sample Size (Percentage of Starting Sample)** | | |
| --- | --- | --- | --- |
|  | **Long-Term Mental Health Problem Not Reported** | **Long-Term Mental Health Problem Reported** | **Combined** |
| **Starting Sample** | 553178 | 57097 | 610275 |
| Less: Labour Force Participation outcome excluded categories | -55504 (10·03%) | -4318 (7·57%) | -59822 (9·80%) |
| **Eligible Sample** | 497674 (89·97%) | 52779 (92·43%) | 550453 (90·2%) |
| Less: missing data |  |  |  |
| Appointments per referral exposure | -9353 (1·69%) | -1056 (1·85%) | -10409 (1·71%) |
| Area-level waiting Times covariate | -2703 (0·49%) | -357 (0·63%) | -3060 (0·5%) |
| Benefits covariate | -854 (0·15%) | -78 (0·13%) | -932 (0·15%) |
| Highest level of qualification covariate | -903 (0·16%) | -81 (0·14%) | -984 (0·16%) |
| **Analysis Sample** | 483861 (87·47%) | 51207 (89·47%) | 535068 (87·68%) |

Table S5 shows that the starting sample for this study comprises 610275 observations, consisting of all individuals aged 18-65 who completed the APS between April 2015 and March 2020. Only English residents are selected for the starting sample as the NHS Talking Therapies data is reported on services provided in England. This study period was chosen as the data used for the NHS Talking Therapies supply measure is consistently reported from 2015, and any APS data after March 2020 is subject to an altered sampling and interview process because of the COVID-19 pandemic. Additionally, we conform the study period to correspond with the NHS data financial year reporting structure (April-March). We exclude any participants that fall under the categories of economic activity as specified in Additional File 2 to obtain the sample of eligible APS participants for our study. We then exclude any individuals with missing exposure and covariate data, giving an analysis sample of 535068 total observations.
