# Supplementary material for: Availability of psychological therapies and workforce participation of individuals with long-term mental health problems: a retrospective observational study
Source: Int J Ment Health Syst. 2026 Apr 15;20:9. doi: 10.1186/s13033-026-00706-z (PMC13200466; doi:10.1186/s13033-026-00706-z)
Supplement: Supplementary file 7 — Supplementary Material 7. [file 13033_2026_706_MOESM7_ESM.docx]

**Additional File 7**

**Figure S1: Regressing the NHSTT Supply Measure on the Adjusted Model Covariates**


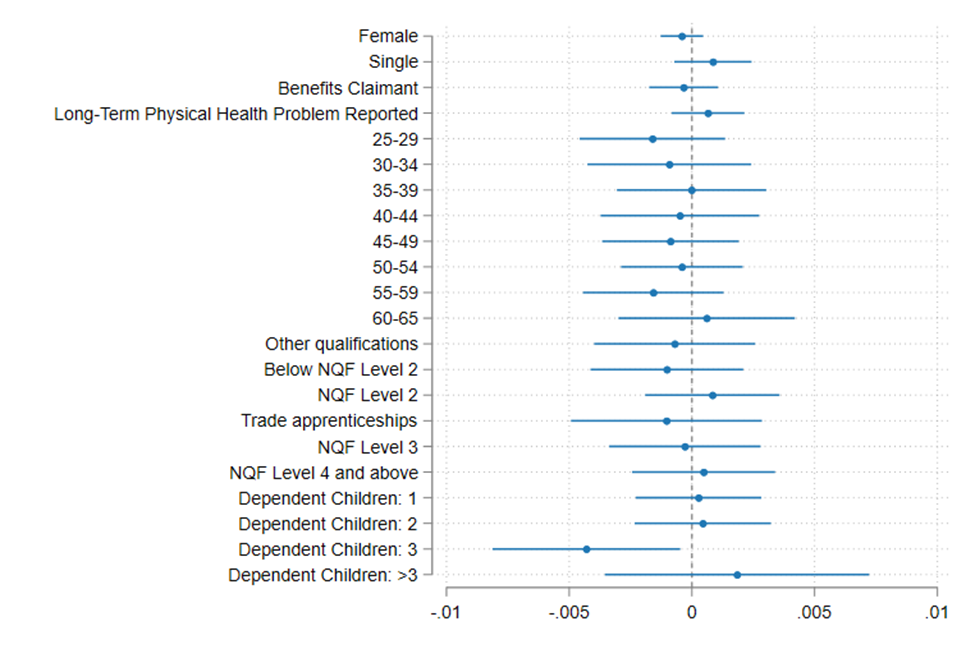
Notes: The regression was estimated on the full analysis sample (N=535068). The coefficient plot includes the following categorical variables: age bands, highest attained level of qualification, and number of dependent children in the household. For age bands, 25-29 through to 60-65 are compared against the base category of 18-24. For qualification level, other qualifications, Below NQF level 2, NQF level 2, trade apprenticeships, NQF level 3, and NQF level 4 and above are compared against the base category of no qualifications. For number of dependent children in the household, 1, 2, 3, and more than 3 are compared against the base level of none.
